# Coming Back to the Basics. Comment on Cangir et al. A CT-Based Radiomic Signature for the Differentiation of Pulmonary Hamartomas from Carcinoid Tumors. *Diagnostics* 2022, *12*, 416

**DOI:** 10.3390/diagnostics13233489

**Published:** 2023-11-21

**Authors:** Armando Perrella, Giulio Bagnacci, Nunzia Di Meglio, Vito Di Martino, Cristiana Bellan, Luca Luzzi, Maria Antonietta Mazzei, Luca Volterrani

**Affiliations:** 1 Unit of Diagnostic Imaging, Department of Medical, Surgical and Neuro Sciences and of Radiological Sciences, University of Siena, Azienda Ospedaliero-Universitaria Senese, 53100 Siena, Italy; giuliobagnacci@gmail.com (G.B.); dimeglionunzia@gmail.com (N.D.M.); dimartino7@student.unisi.it (V.D.M.); mariaantonietta.mazzei@unisi.it (M.A.M.); lucavolterrani@gmail.com (L.V.); 2 Unit of Pathological Anatomy and Histology, Department of Medical Biotechnologies, University of Siena, Azienda Ospedaliero-Universitaria Senese, 53100 Siena, Italy; cristiana.bellan@unisi.it; 3 Thoracic Surgery Unit, Department of Medical, Surgical and Neuro Sciences, University of Siena, Azienda Ospedaliero-Universitaria Senese, 53100 Siena, Italy; luca.luzzi@unisi.it

We read with great interest the article by Cangir et al., “A CT-Based Radiomic Signature for the Differentiation of Pulmonary Hamartomas from Carcinoid Tumors”, published on 5 February 2022 [1].

This interesting study demonstrated the benefits of radiomics analysis in the differentiation between hamartomas (HAs) and neuroendocrine neoplasms (NENs) of the lung, two extremely different entities, which can sometimes be morphologically indistinguishable. While HAs are invariably benign lesions, NENs are considered malignant neoplasms, even if well-differentiated or typical NENs, and, therefore, have different clinical management, and the patient must be evaluated for surgical excision [2]. In this manuscript, the authors prepared two radiomics hand-crafted models: (a) the first model included all the data regarding the 138 patients to differentiate between the 78 NENs and 60 HAs; (b) the second model included 78 NENs and 38 HAs without signs of fat tissue. The results revealed that the random forest multilayer perceptron method (RF-MLP) was the best for the HAs (area under the curve—AUC = 0.999) and NENs (AUC = 0.999) for the first model (AUC = 0.836) and PCT (AUC = 0.836) in the test set for the second model. The authors also found that eight radiomics features could differentiate between NENs as HAs, namely the maximum two-dimensional diameter (row), maximum two-dimensional diameter (column), long-run low gray level emphasis (LRLGLE), the dependence variance (DV), kurtosis, large-dependence low gray level emphasis (LDLGLE), and maximum three-dimensional diameter. 

Even if those results are interesting, it is known in the literature that the diagnosis of solitary pulmonary nodules may be possible via the analysis of post-contrastographic features [3]. For example, in our study [4,5], we demonstrated the possibility of distinguishing perfectly pulmonary HAs and NENs via simple biphasic contrast-enhanced CT (CECT). Between September 2015 and December 2021, 95 patients with a histologically proven diagnosis of lung NENs (74) and HAs (21) and who underwent a preoperative CECT scan were initially identified via a review of our pathological and radiologic databases. Among these, 55 cases were reviewed by three radiologists with different levels of experience, analyzing their morphologic and enhancement features. The enhancement analysis was performed by placing a region of interest (ROI) within the lesion in non-contrast (NCp), post-contrast (PCp, 55 to 65 s after intravenous contrast injection), and delayed phases (Dp, 180 to 300 s after intravenous contrast injection). HU values were significantly different between NENs (99.1 ± 38.3 HU) and HAs (34.1 ± 18.9 HU) in PCp (*p* < 0.001). NCp and Dp HU attenuation values did not show significant differences in the two groups (19.4 ± 15.1 HU versus 16.6 ± 9.5 HU on NCp and 54.8 ± 19.5 HU versus 50.2 ± 22.1 HU on Dp, *p* > 0.05), but the differences in the values of HUs in PCp and Dp allowed us to perfectly discriminate between NENs and Has; indeed, with our wash-out analysis, ΔHU(PCp-Dp), we could perfectly discriminate pulmonary “fat-poor” HAs from NENs (44.2 ± 28 HU vs. −16.1 ± 8.4 HU, *p* < 0.001). This comment should not be seen as an attempt to belittle the authors’ remarks, which are very important and deserve to be promulgated. On the contrary, considering our results could be useful to evaluate the possibility of including the washout analysis, or it might be an additional hypothesis to study. Although artificial intelligence has demonstrated capabilities and can help radiologists detect or characterize lung nodules [1,5], we must should not forget the basics. In the era of radiomics, dual-energy CT (DECT), and artificial intelligence, radiologists are facing difficulties of evolving by learning new skills, coming back to our basics, and discovering the possibility of achieving a difficult diagnosis with a strict but simple old method can be a nice confidence boost. Moreover, it can be especially useful in those spoke centers that do not have easy access to DECT or processing software for perfusion analysis or radiomics.
